# Assessing Dispersal Patterns of Fish Propagules from an Effective Mediterranean Marine Protected Area

**DOI:** 10.1371/journal.pone.0052108

**Published:** 2012-12-20

**Authors:** Antonio Di Franco, Giovanni Coppini, José Martin Pujolar, Giulio A. De Leo, Marino Gatto, Vladyslav Lyubartsev, Paco Melià, Lorenzo Zane, Paolo Guidetti

**Affiliations:** 1 Laboratory of Conservation and Management of Marine and Coastal Resources, Dipartimento di Scienze e Tecnologie Biologiche ed Ambientali, University of Salento-Consorzio Nazionale Interuniversitario per le Scienze del Mare, Lecce, Italy; 2 Laboratoire ECOMERS, Université de Nice Sophia-Antipolis, Faculté des Sciences, Nice, France; 3 Istituto Nazionale di Geofisica e Vulcanologia, Bologna, Italy; 4 Department of Biology, University of Padova, Padova, Italy; 5 Department of Environmental Science, University of Parma, Parma, Italy; 6 Hopkins Marine Station, Stanford University, Pacific Grove, California, United States of America; 7 Dipartimento di Elettronica e Informazione, Politecnico di Milano, Milano, Italy; 8 Centro Euro-Mediterraneo per i Cambiamenti Climatici, Bologna, Italy; Leibniz Center for Tropical Marine Ecology, Germany

## Abstract

Successfully enforced marine protected areas (MPAs) have been widely demonstrated to allow, within their boundaries, the recovery of exploited species and beyond their boundaries, the spillover of juvenile and adult fish. Little evidence is available about the so-called ‘recruitment subsidy’, the augmented production of propagules (i.e. eggs and larvae) due to the increased abundance of large-sized spawners hosted within effective MPAs. Once emitted, propagules can be locally retained and/or exported elsewhere. Patterns of propagule retention and/or export from MPAs have been little investigated, especially in the Mediterranean. This study investigated the potential for propagule production and retention/export from a Mediterranean MPA (Torre Guaceto, SW Adriatic Sea) using the white sea bream, *Diplodus sargus sargus*, as a model species. A multidisciplinary approach was used combining 1) spatial distribution patterns of individuals (post-settlers and adults) assessed through visual census within Torre Guaceto MPA and in northern and southern unprotected areas, 2) Lagrangian simulations of dispersal based on an oceanographic model of the region and data on early life-history traits of the species (spawning date, pelagic larval duration) and 3) a preliminary genetic study using microsatellite loci. Results show that the MPA hosts higher densities of larger-sized spawners than outside areas, potentially guaranteeing higher propagule production. Model simulations and field observation suggest that larval retention within and long-distance dispersal across MPA boundaries allow the replenishment of the MPA and of exploited populations up to 100 km down-current (southward) from the MPA. This pattern partially agrees with the high genetic homogeneity found in the entire study area (no differences in genetic composition and diversity indices), suggesting a high gene flow. By contributing to a better understanding of propagule dispersal patterns, these findings provide crucial information for the design of MPAs and MPA networks effective to replenish fish stocks and enhance fisheries in unprotected areas.

## Introduction

Many studies have highlighted the positive effects of successfully enforced marine protected areas (hereinafter MPAs) on populations of exploited coastal fishes in both tropical and temperate areas [1]–[3]. On a global scale, these studies have shown that density, biomass and size of exploited species tend to increase under protected conditions (see [4], [5], [6], [7] and references therein).

Due to a number of processes (e.g. density-dependent effects, ontogenetic migrations and diffusive movements), MPAs may also provide benefits to outer areas through spillover of juvenile and adult fish (see [8] for a review). There is an increasing body of scientific evidence suggesting that spillover of mobile adults from MPAs may replenish nearby exploited populations and therefore enhance fisheries adjacent to MPAs [9]. An additional process leading to population replenishment and fisheries enhancement in outer areas is the so-called ‘recruitment subsidy’ (following [8], see also references therein), i.e. the export of propagules (eggs and larvae) from MPAs.

MPAs hosting a high density of spawners (large-sized, mature individuals) have the potential to increase the occurrence of spawning aggregations (i.e. high-density groupings of conspecific fish gathered together for the purpose of reproducing) and, more in general, to generate a greater propagule production compared to fished areas [10], [11]. Therefore, besides the potential to enhance adjacent fisheries through spillover, some modeling studies suggest that MPAs can produce propagules that could be retained and/or exported outside their boundaries, even towards sites located at quite large distance [12], [13]. From this perspective, the use of MPAs as a way for improving ecosystem-based management has been widely advocated as crucial and complementary to large-scale spatial planning [14], [15].

While there is an abundant literature reporting cases of fish population recovery inside MPAs [16], a scarce body of evidence is available about the actual role of MPAs in sustaining fish stocks in fished areas beyond MPAs’ borders through propagule production and export [17]. Determining whether and how this process actually takes place is considered one of the major research gaps in MPA science [18]. A pre-condition for the increase in propagule production and the consequent retention/export from an effective MPA is the recovery of population abundance and structure within the MPA compared to outside. When this happens, the benefits of protection within the MPA have the potential to extend also well beyond its boundaries through larval dispersal and settlement support, highlighting the need to have well-enforced MPAs [19].

Empirical estimates of propagule retention/export are rare, due to the difficulties associated with tracking small-sized fish propagules from their natal origins through the pelagic environment to their possible settlement locations [20]–[24]. Direct methods for assessing propagule retention/export are also hindered by the dramatically low concentration of propagules in the open waters, while indirect approaches, such as microchemistry and genetic methods, have just begun to adequately describe spatial patterns of connectivity [20], [25]–[26]. Modeling approaches, conversely, have been widely used to investigate dispersal patterns and provide useful scenarios for management [27]–[32]. In particular, Lagrangian (i.e. individual-based) models allow the integration of physical hydrodynamic models with data on key biological traits. Biological information is typically represented by the timing of placement of eggs and/or larvae (i.e. spawning dates for fishes) and the time spent in the plankton (i.e. pelagic larval duration, hereinafter PLD), that, along with information about swimming speed and patterns of vertical migration, may help refine estimates of dispersal patterns [32], [33].

Most Lagrangian model applications are based on PLD values and spawning dates from single estimates (in space and in time) that have been extrapolated over larger scales. This is understandable due to the effort (time and resource consumption) and specific competences (e.g. ability to analyze otolith microstructure) required to get reliable information on PLD and spawning or birth dates. There is increasing evidence, however, that these biological traits significantly change in time and space [34]–[36], suggesting the need to use spatially and temporally proper (i.e. contextual) data to refine local predictions on dispersal patterns of propagules. In addition, studies aimed at assessing potential retention/export of propagules from MPAs on the basis of dispersal models usually lack supporting field-based evidence. Only very few studies, in fact, have investigated spatial and/or temporal patterns of settlement in supplying MPAs or nearby fished areas, strongly suggesting propagule export of mollusks and fishes [31], [37], [38]. Nevertheless, empirical measures of larval dispersal are still largely missing (but see [22]–[24]). While measuring propagule production and abundance is often difficult, settlement of newly metamorphosed larvae at appropriate habitats, which reflects abundance patterns of successfully settled larvae, can be quite easily investigated [39].

Information on patterns of spatial connectivity of fish populations can be obtained using a number of approaches (e.g. field data, modeling, genetic patterns; [20]) that can contribute to a better understanding of propagule retention and/or export from MPAs. This information is essential for the design of single MPAs and of MPA networks.

The aim of the present work is to investigate the potential propagule production and retention/export of a coastal fish from a Mediterranean MPA (Torre Guaceto MPA, SW Adriatic Sea). The white sea bream (*Diplodus sargus sargus,* Linnaeus 1758) was selected as a model species due to its ecological and economic importance [40], [41]. The originality of this study is the use of a multidisciplinary approach combining field observations, modeling of larval dispersal and molecular genetics to: 1) assess population recovery inside the MPA, 2) simulate patterns of retention and export of propagules from the MPA, 3) explore the effects of potential retention/export from the MPA on settlement patterns and on the genetic structure of the population(s) studied.

## Methods

### Study Area and Species

**Figure 1:**
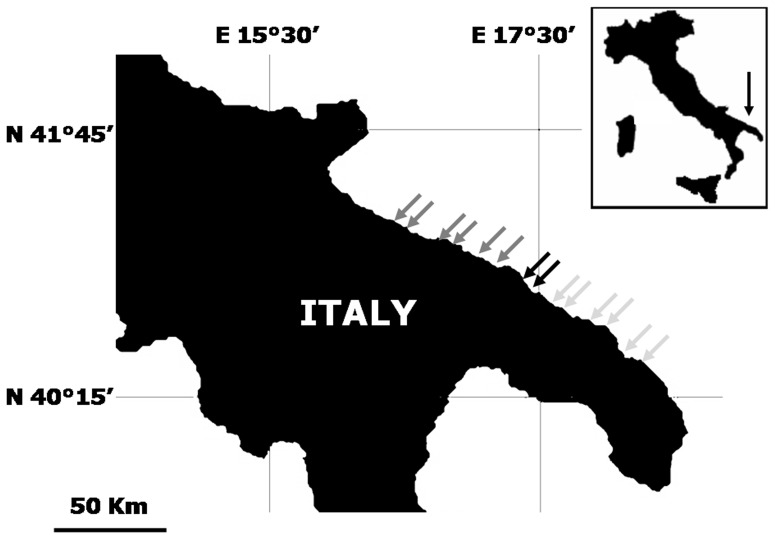
Study area. Arrows indicate the sampling sites. Black arrows indicate sites within TGMPA, dark grey arrows indicate sites north of TGMPA borders and light grey arrows indicate sites south of TGMPA borders. See Table 1 for name and coordinates of the sampling sites.

The study was carried out at Torre Guaceto MPA (hereinafter TGMPA, 40°42′N; 17°47′E) and in surrounding unprotected areas up to about 100 km away from TGMPA borders. TGMPA is located in southeastern Italy, along the Apulian Adriatic coast (Mediterranean Sea; Fig. 1, Table 1). It was formally established in 1991, but enforcement became effective around 2000–2001 due to the previous shortage of MPA personnel and surveillance from maritime authorities (e.g. coast guard). The entire MPA covers 2227 ha, stretching along about 8 km of coastline, and is subdivided into three zones: (1) a no-take/no-access reserve (zone A, 179 ha); (2) a general reserve (zone B, 163 ha) and (3) a partial reserve (zone C, 1885 ha), where restrictions to human activities become progressively less severe. Access to zone A is restricted to scientists, reserve personnel and police authorities. In zone B only recreational bathing from the coast is allowed. In zone C, both professional and recreational fishing are allowed subject to permission of TGMPA management body, with the exception of spearfishing. Outside the TGMPA, fishing regulations are less restrictive compared to within the MPA (e.g. spearfishing is allowed, and recreational and professional fishing are regulated by national laws). The TGMPA is effectively enforced [19] and this is demonstrated by the clear ‘reserve effect’ observed at population and community levels [40].

**Table 1:** Details of sampling sites within each Geographic Area.

Geographic Area	Site Name	LAT N	LONG E
**North**	Torre a Mare	41.084236°	17.025161°
	Bari San Giorgio	41.095375°	16.974892°
	Porto Marzano	40.930019°	17.332558°
	Cala Corvina	40.980203°	17.259275°
	Torre Pozzella	40.770125°	17.668933°
	Hotel La Darsena	40.776886°	17.644958°
**TGMPA**	Terza Baia	40.717703°	17.788128°
	Punta Penna Grossa	40.727228°	17.761078°
**South**	Torre Rossa	40.686767°	17.868042°
	Punta Penne	40.682494°	17.934994°
	Casalabate	40.497744°	18.123053°
	Torre Rinalda	40.473989°	18.176017°
	San Foca	40.297436°	18.413506°
	Conca Specchiulla	40.244933°	18.450475°

The climatological circulation pattern of the Southern Adriatic Sea, where the study area is located, is characterized by well-known current and gyre structures [42]–[45], such as the cyclonic Southern Adriatic (SAd) gyre [42] located in the southern sub-basin. The SAd gyre is interconnected (with seasonally varying characteristics) with the Middle Adriatic (MAd) gyre (located in the central sub-basin) by two coastal currents: one flowing southward along the whole western coast from the Po delta to the Otranto Strait (Western Adriatic Coastal Current or WACC), the other flowing northward from the Otranto Strait along the eastern coast and reaching the central Adriatic sub-basin (Eastern Southern Adriatic Current or ESAC, [45]).

The white sea bream, *Diplodus sargus sargus* (Linnaeus 1758), usually inhabits the littoral zone in shallow waters down to about 50 m [46], [47]. It is targeted by many professional and recreational fisheries, and consequently shows a clear increase in density and size when protected from fishing [40], [48], [49]. Adults are relatively sedentary [50] and demersal, and produce eggs and larvae that develop in pelagic waters during a period ranging from 14 to 28 days [34], [35], [51]. Then post-larvae metamorphose and settle at about 1 cm total length (TL) in shallow (<2 m depth) coastal benthic habitats, mainly small bays with mixed sand and rocky bottom [47], [52]–[54].

### Sampling Design, Data Collection and Statistical Analysis

Density, size distribution and biomass of adults (i.e. reproductive individuals >18 cm, [55]), and density of settlers (i.e. specimens <1.5 cm) were assessed at fourteen sites along approximately 200 km of the Apulian Coast in the South Adriatic Sea (Fig. 1, see Table 1 for names and coordinates of sampling sites). Two sites were placed inside the Torre Guaceto Marine Protected Area (TGMPA) and twelve sites were outside (six northwards and six southwards) in surrounding unprotected areas up to ∼100 km from the MPA.

Each site was defined as a stretch of coastline 10–100 m long. At each site, a total of 24 replicated visual censuses were carried out (n = 16 for settlers, n = 8 for adults) for a total of 336 replicates. Due to the high number of replicates, the sampling for settlers was carried out by two operators equally partitioning the number of replicates within each site.

Density and size of adults were estimated in July 2010 by scuba underwater visual census using strip transects of 25×5 m [56] at 6–10 m depth. In each transect, abundance and size of fish encountered were recorded. Fish size (total length, TL) was recorded within 2 cm size classes. Fish wet-weight was estimated from size data by means of a length–weight relationship available from the literature and referring to Mediterranean samples [57].

Density of settlers was evaluated during the settlement peak in June 2009 by means of snorkeling visual census along strip transects of 25×2 m [53], [56] at 0–2 m depth. For both adults and settlers each transect was explored in ∼10 min. Actual number of fish encountered was recorded up to 10 individuals, whereas larger groups were recorded using categories of abundance (i.e. 11–30, 31–50, 51–200, 201–500, >500 ind.; see [56]). Transects were parallel to the coastline, in small embayments (i.e. 200–400 m) with shallow rocky habitats alternated with sand patches, which represent the preferential habitat for settlers of *D. sargus sargus* [46], [47], [53].

Sampling sites were randomly selected out of a pool of possible sites having similar features in terms of habitat types’ coverage and exposition.

Concomitantly with density estimates, settlers of *D. sargus sargus* were collected for a preliminary genetic analysis. Settlers were collected in June 2009 at three separate sites within the study area: (i) one site (Punta Penna Grossa) within the TGMPA, (ii) one site (Torre Pozzella) located ∼20 km north of the MPA, and (iii) one site (Punta Penne) located ∼15 km south of the MPA. Genetic analyses were conducted on a sub-sample of 96 individuals, including 48 from the MPA, 24 from Torre Pozzella and 24 from Punta Penne. After collection both post-settlers and recruits were immersed in an ice slurry (<5°C) to minimize suffering and then stored in 95% ethanol. The experimental fishing activity was performed in strict accordance with the authorization protocol provided by the Italian Ministry of Agriculture, Foods and Forestry Politics (Permit Number: 0011267–2010).

To test for potential differences in fish densities along the study area, two univariate PERMANOVA analyses were carried out separately for adults and settlers. An asymmetrical sampling design was adopted that included the following factors: Geographic Area (GA; Fixed factor with three levels: North, MPA, South) and Site (Si, random factor, nested within GA, with 2–6 levels: 2 in TGMPA, 6 in South and North). Linear distance (Di) in km from TGMPA boundary (set to 0 for the two sites within TGMPA) was used as a covariate. Distances were measured using georeferenced satellite imagery from Google Earth (http://earth.google.com).

The same experimental design was adopted to test for potential spatial variation in biomass of adult specimens. Post-hoc pairwise tests were carried out, whenever appropriate, if PERMANOVA detected significant differences.

Putative differences in adult size among GAs were tested using 1-way PERMANOVA, which does not involve any assumption about the distribution of the variable. For each GA, individual fish size data were pooled and plotted as size–frequency distributions. Statistical analyses were run using Primer 6 PERMANOVA+software package (Plymouth Marine Laboratory).

### Adriatic Forecasting System and Lagrangian Simulations

The study area is covered by the Adriatic Forecasting System (AFS) which provides daily oceanographic model outputs consisting of simulation (for the past time) and forecast for the next 10 days [45], [58]. The output products of the model are daily fields of ocean temperature, salinity and current on a regular three-dimensional grid. AFS products are produced and delivered by Istituto Nazionale di Geofisica and Vulcanologia (INGV, http://gnoo.bo.ingv.it/afs).

The model used is the POM (Princeton Ocean Model). It is a three-dimensional finite difference, free surface, primitive equation numerical model, based on the Boussinesq and the hydrostatic approximation and a split mode time step. The AFS model domain encompasses the whole Adriatic basin and extends south of the Otranto channel into the northern Ionian Sea, where the only open boundary is located. The AREG (Adriatic REGional model) grid has a horizontal resolution of about 1/45° (about 2.2 km), on 31 σ-layers. The surface fluxes are interactively computed using sea surface temperature predicted by the model and realistic atmospheric data provided by the European Centre for Medium Range Weather Forecast (ECMWF) with a frequency of six hours and a resolution of 0.25°. AFS is nested into the Mediterranean Forecasting System, MFS [59], [60] that provides to AFS the initial and lateral boundary conditions for temperature, salinity and velocity. The lateral boundary conditions are taken from MFS on a daily basis. In MFS the use of the data assimilation [61] allows the reconstruction of the ocean dynamics in the past by merging model simulations with satellite and *in situ* observations.

The Lagrangian simulations used in this work were performed by using the User Visualization Tool (UVT), a software developed by INGV that ingests the AFS model products allowing the user to derive particle trajectories by simulation. Each Particle (P), representing a single propagule, is described in terms of its spatial position (P_x,y,z_) at each time step (t). The depth at which the particle is released at the beginning of the simulation (t = 0) was kept constant along the entire simulation as quantitative data is not available to support alternative assumption on larval behavior and vertical movements for the studied species.

UVT calculates current velocity components (u_i,j_,v_i,j_) for each particle location (P_i,j_) at time t (spatial and temporal interpolation of AFS model output) using the information provided at each grid point by AFS products. UVT then calculates the movement of the particle ((u_i,j_,v_i,j_)×dt) where dt corresponds to the time step of integration. The new position of the particle at t+dt is then calculated. The horizontal resolution of the AFS model grid does not allow a precise simulation of hydrodynamic processes very close to the coast, and this should be taken into account when interpreting simulated dispersal patterns.

Results from otolith analysis taken from [34] provided information on the spawning schedule and PLD. Passive particles were released in UVT within TGMPA and tracked for 17 days (the average PLD estimated through otolith analysis; [34]) in order to assess dispersal patterns from TGMPA. Simulations were carried out at three different depths (1, 5 and 10 meters) along the water column. These depths were selected based on the available information on vertical distribution of white sea bream propagules, indicating that larvae are more frequently collected within 0–10 meters [62]. Twenty-four particles were released during each simulation. The number of particles was set up after preliminary analyses that did not highlight any detectable difference in trajectories increasing the number of particles to over 24 (Coppini and Lyubartsev, data not reported). The number of particles, therefore, was chosen in order to allow a clear visualization of trajectories of tracers.

The starting positions of the particles were defined over an equally spaced grid within a circle of 5 km around the actual sampling position. Simulations were started in coincidence of 4 spawning dates that were back-calculated from otolith analysis [34] - namely 9^th^, 11^th^, 13^th^, 15^th^ of May, 2009– and were aimed at representing the oceanographic regime occurring at the time of, and in the 17 days following, the spawning events. A total of 12 simulations (arising from the combination of 4 spawning dates and 3 depths: 1 m, 5 m and 10 m) were performed. High consistency among repeated simulations (i.e. two simulations carried out under the same experimental setting) was observed during preliminary analyses.

### Genetic Analyses

Minute sections of the caudal fin were digested in a lysis buffer containing 100 µl TE Buffer, 7 µl 1 M DTT (dithiothreitol) solution pH 5.2 (diluted in 0.08 M NaAC) and 2 µl proteinase K solution (20 mg/ml) for at least 8 hours at 56°C. After incubation at 96°C for 10 min, samples were centrifuged at 13,000 rpm for 11 min, and the supernatant was stored at −20°C.

Genotypes were examined at a total of 12 microsatellite loci originally developed for the gilthead sea bream *Sparus aurata* [63], [64] that positively amplified in *D. sargus sargus*. Microsatellites were grouped into two separate multiplexes in order to reduce PCR and genotyping costs (Table 2). PCR products were obtained in a GeneAmp PCR System 2700 Thermocycler (Applied Biosystems) using the QIAGEN Multiplex PCR Kit. PCR reactions consisted of 2 µl template DNA, 5 µl QIAGEN Multiplex PCR Master Mix, 0.2 µl 10 µM forward and reverse primers and water up to 10 µl. PCR conditions were as follows: 15 min at 95°C, 35 cycles of 30 sec at 94°C, 90 sec at 57°C and 1 min at 72°C and final elongation for 30 min at 60°C. PCR products were visualized on 1.8% agarose gels and screened for microsatellite polymorphism using an ABI 3130 AVANT automatic capillary sequencer (Applied Biosystems).

**Table 2:** Primers used in the genetic study of *Diplodus sargus sargus*.

Locus	Acc. No.	Reference	Forward and Reverse primer	Motif	TNA	Size	MX
Ad05	DQ851244	[63]	TGATCACACACTCTATACAGGCTC GTGTGCCATTTTTCCTCCA	AC	28	132–187	2
Ad86	DQ851248	[63]	TTCTCCCTCCTCTCCACTCA CCTGCTTTCTCTATGCCTCG	AC	18	159–199	2
Bd09	DQ851277	[63]	CCAGGGAGAGCTCTTCATCTT GCGTTAAATGCATAAACAGCTAAG	AC	13	101–125	1
Bld15	DQ851279	[63]	CACCAATCACTCGGCTTCAC GCAGCTAAAAGCTACTGGGAGA	TG	29	161–222	1
Bld39	DQ851282	[63]	CTCCTGCTGCTGAAACTCCT GCATAGCGCGTACCAAAAGT	TG	11	209–231	1
Cld11	DQ851303	[63]	TACCGCTGCAGATGTGAGTC AACACCTCCTGTCATGTTTGC	AC	4	196–202	2
Cld26	DQ851307	[63]	CTTCTGCTGGTGTTTGTTTCTG CACAAACCAGTTCAACAAGAGC	AC	27	177–222	1
Cld32	DQ851310	[63]	GGCGTCTTGTCTGACTGCAT GCTGTCATCTGTAAGCTGCAT	AC	15	141–169	1
Dld14	DQ851338	[63]	AGATTGGCCTGTGATCCTTG GTACAAACCATCCCGCTGTC	AC	24	95–141	2
Dld31	DQ851345	[63]	CATGGGACCAGTGGGAACTA TCATTTGGGGCTCTCATTTC	AC	5	140–148	1
Cld49	FN811860	[64]	ACCTCGTCAGCGATCCATAC GGCTGCCACTAGTTTTCTGC	AC	24	72–120	1
Cld93	FN811861	[64]	CCCAACAACCCGTTCCAG CACCTGGGTCATTAGCTGTG	TG	4	188–196	2

Details include Genbank accession number (Acc. No.), repeat motif, total number of alleles detected (TNA), allele size range for all loci and multiplex in which all loci were included (MX).

Within sample genetic diversity was assessed by observed (*H*_o_) and expected (*H*_e_) heterozygosities per locus using GENETIX version 4.05 [65] and allelic richness (AR) using FSTAT version 2.3.9.2 [66]. Diversity values across samples were compared with one-way ANOVA using STATISTICA version 10 (StatSoft). Deviations from Hardy-Weinberg equilibrium (HWE) and linkage disequilibrium were tested using GENEPOP version 3.4 [67]. Significance levels for multiple comparisons were adjusted using the sequential Bonferroni technique [68]. Presence of null alleles was tested using MICROCHECKER version 2.2.3 [69].

Prior to the population structure analysis, the statistical power of the markers employed was assessed with POWSIM [70]. We tested a range of predefined levels of expected divergence (F_ST_ = 0.001, 0.005, 0.01, 0.05, 0.1) using an N_e_ value of 1,000. Differences in allele and genotype frequencies among samples were assessed using Fisher's exact test as implemented in GENEPOP. Significance levels for multiple simultaneous comparisons were adjusted using Bonferroni as described above. Population structure was explored by nonhierarchical AMOVA and by calculating pairwise F_ST_ between samples in ARLEQUIN 3.5.1.2 [71]. Population substructuring was also explored with the software STRUCTURE [72], a model-based clustering algorithm that infers the most likely number of groups in the data. The software organizes individuals into a predefined number of clusters (K) with a given likelihood, which might represent putative populations. The analysis was performed for 1< K <3, with five replicates per K and using the admixture model. A burn-in length of 10^4^ iterations followed by 10^6^ additional Markov Chain Monte Carlo (MCMC) iterations were performed. The most likely K was determined using the criterion of [73].

## Results

### Spatial Distribution Patterns of Adults and Settlers

**Figure 2:**
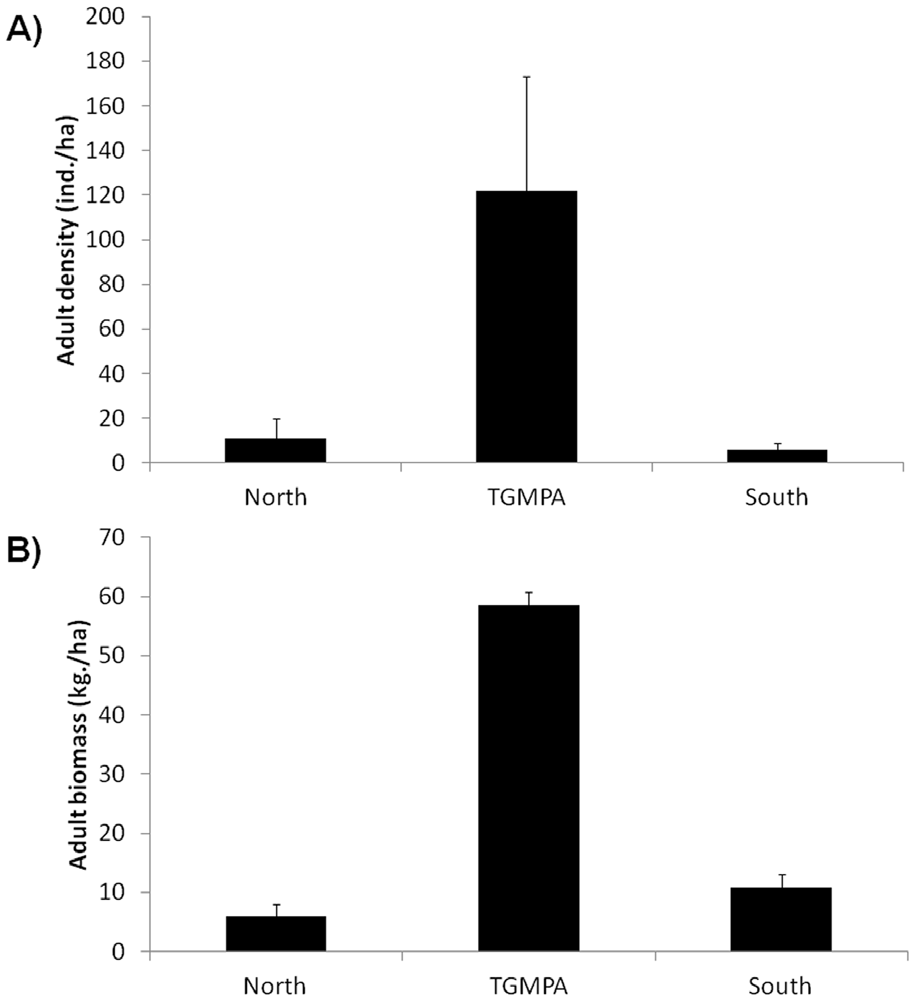
Mean (± SE) density (panel A) and biomass (panel B) of adult white sea bream assessed at the three Geographic Areas.

Density of adult *D. sargus sargus* was significantly affected by distance from TGMPA, i.e. it decreased with distance from TGMPA borders. Within TGMPA density was significantly higher than that observed at both northern and southern GAs (122±51, 11±9 and 6±3 ind./ha respectively; Pairwise tests: TGMPA>North = South, Fig. 2). Density significantly varied among sites (Table 3).

**Table 3:** PERMANOVA on density and biomass data of adult specimens.

		Density		Biomass	
Source	d.f.	MS	Pseudo- f	MS	Pseudo- f
Di	2	21.16	4.81*	1.97E6	42.48***
GA	4	13.96	3.19**	2.04E6	44.07***
Si	7	4.37	2.54***	46396	0.23 ns
Res	98	1.71		2.01E5	
Total	111				

ns: not significant; *p<0.05; **p<0.01; ***p<0.001. Di = Distance, GA = Geographic area, Si = Site.

**Figure 3:**
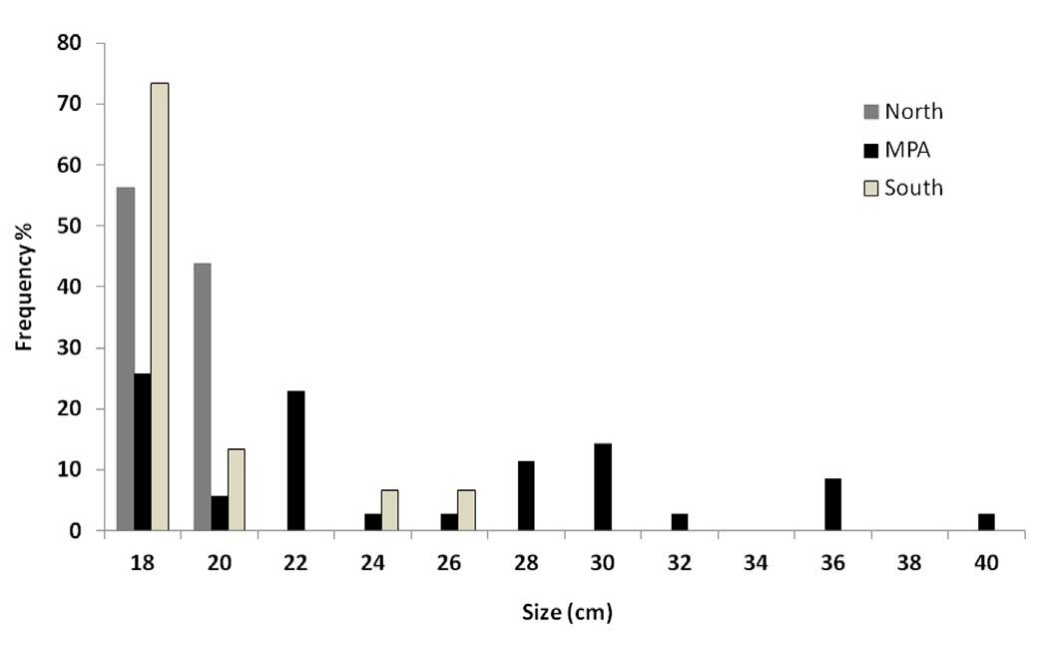
Size frequency distribution of adult white sea bream in the three different Geographic Areas.

Significant differences in average body size were found among GAs (p<0.001, Fig. 3). Pairwise tests highlighted that bigger fish sizes were observed within TGMPA compared to South and North areas (p<0.001 for both pairwise tests) while no significant difference was detected between South and North.

Biomass of adult white sea bream was related to distance from TGMPA (with density decreasing at increasing distance from TGMPA borders) and it was significantly higher at TGMPA than at northern and southern areas (58.5±2.3, 6±2.4 and 10.9±2.2 Kg/ha; pairwise tests: MPA>North = South, Fig. 2). Adult biomass did not vary significantly at the spatial scale of sites (Table 3).

**Figure 4:**
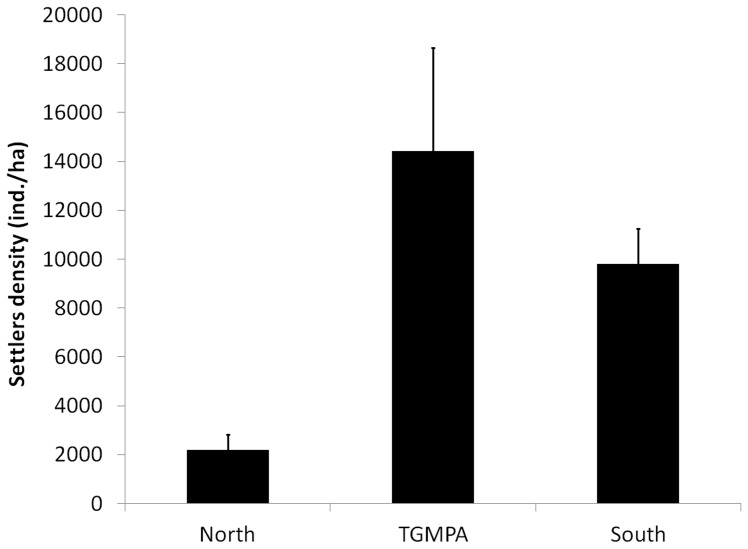
Mean density (± SE) of settlers of white sea bream assessed at the three Geographic Areas.

Density of settlers was not related to distance from TGMPA and was significantly affected by GA. The highest density of settlers was found within the TGMPA (14431.25±4205 ind./ha, mean±S.E.), followed by that observed at the southern GA (9808.33±1438.75 ind./ha), while the lowest values were found at the northern GA (2202.08±624.65 ind./ha). Mean density of settlers at both TGMPA and southern GA were significantly greater than the mean values in northern GA (pairwise tests: TGMPA = South>North, Fig. 4). Density of settlers significantly differed among sites (Table 4).

**Table 4:** PERMANOVA on density data of settlers.

		Density	
Source	d.f.	MS	Pseudo- f
Di	1	5531.8	0.49 ns
GA	2	59920	5.32*
Si	10	11255	2.69***
Res	210	4188.7	
Total	223		

ns: not significant; *p<0.05; ***p<0.001. Di = Distance, GA = Geographic area, Si = Site.

### Simulated Dispersal Trajectories and Travel Distances

**Figure 5:**
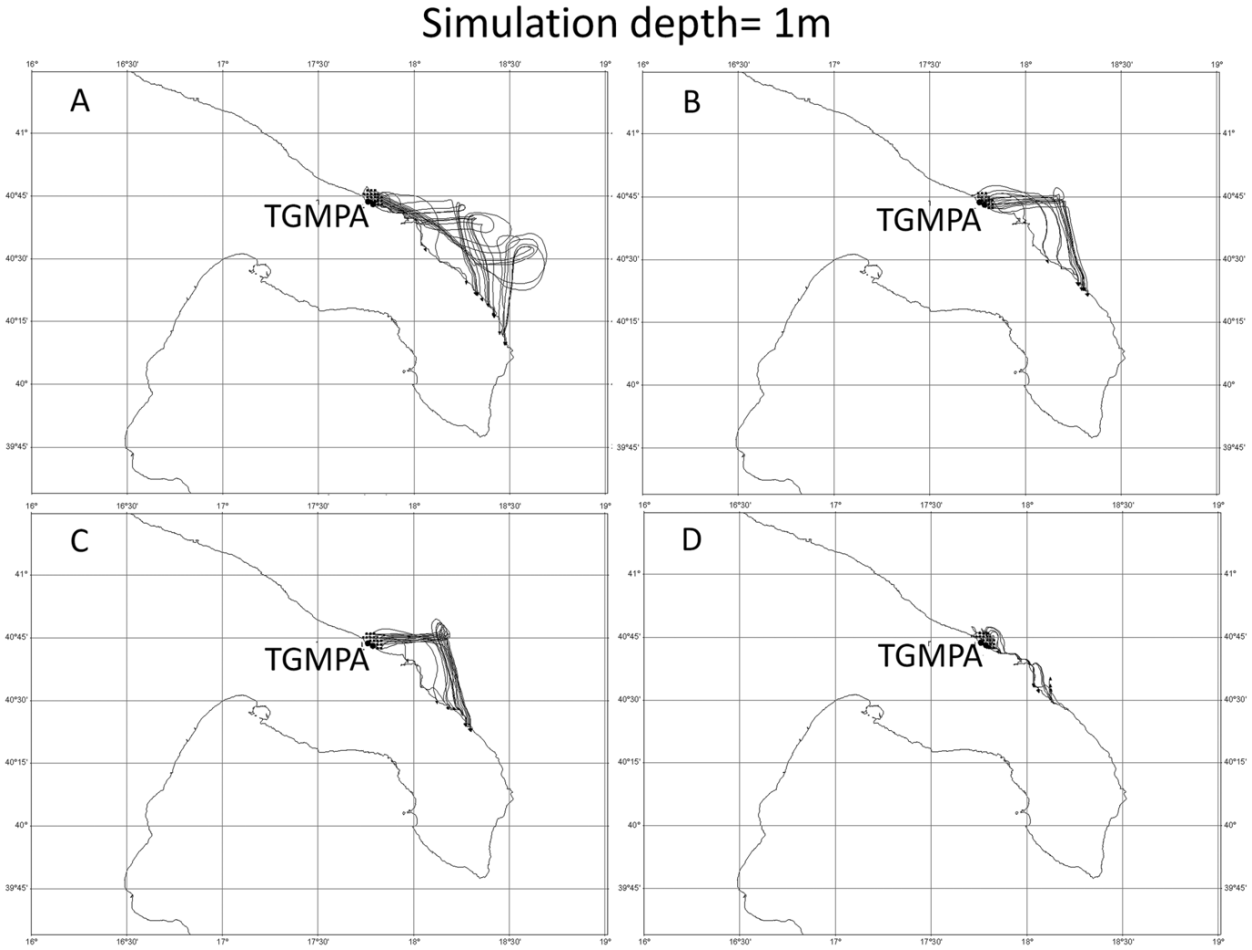
Four Lagrangian simulations at 1 meter depth. Simulations started respectively a) on 9^th^, b) 11^th^, c) 13^th^, d) 15^th^ of May. All simulations lasted 17 days.

**Figure 6:**
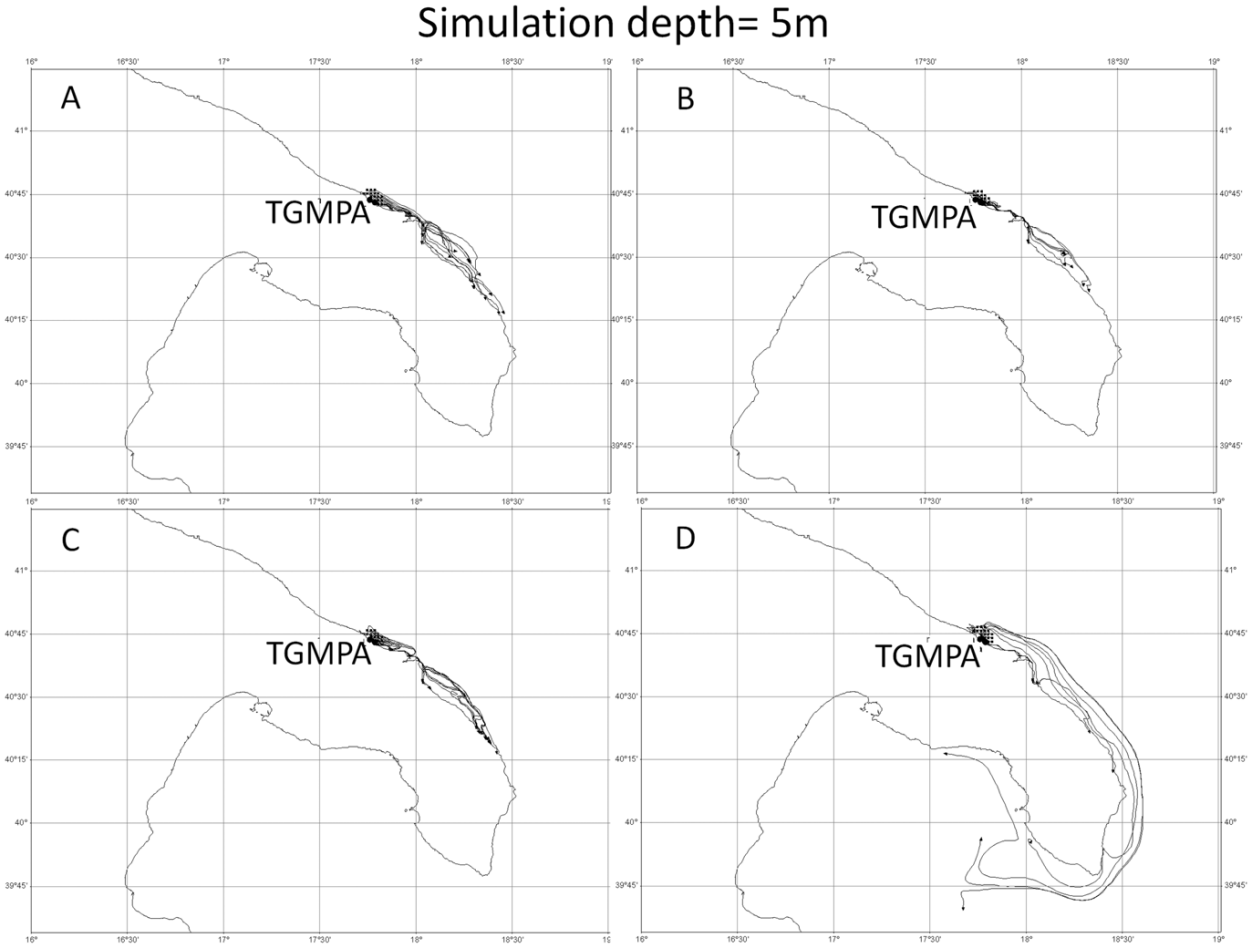
Four Lagrangian simulations at 5 meter depth. Simulations started respectively a) on 9^th^, b) 11^th^, c) 13^th^, d) 15^th^ of May. All simulations lasted 17 days.

**Figure 7:**
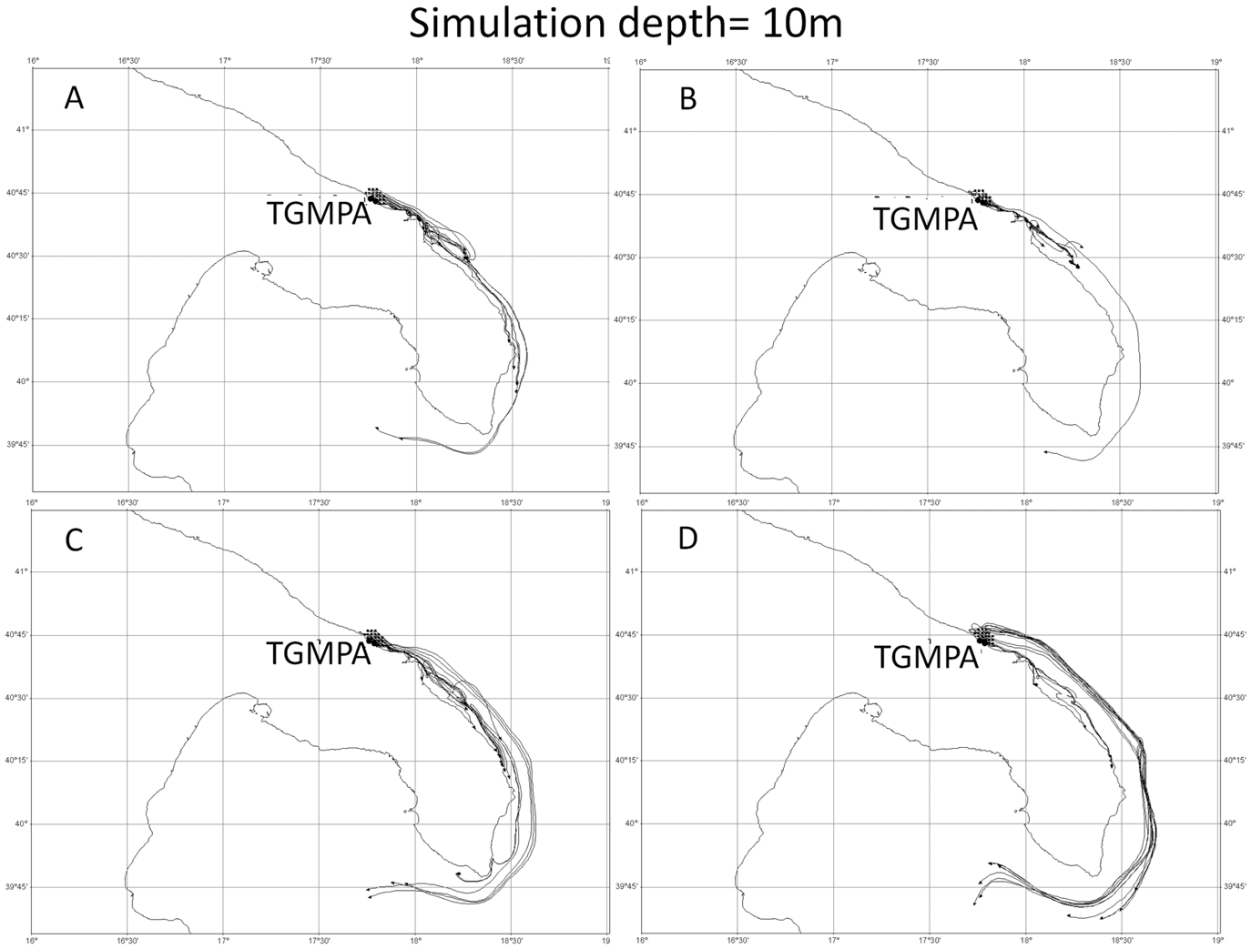
Four Lagrangian simulations at 10 meter depth. Simulations started respectively a) on 9^th^, b) 11^th^, c) 13^th^, d) 15^th^ of May. All simulations lasted 17 days.

The twelve simulations (4 release dates×3 depths) generated with the UVT showed that the particles released from TGMPA stayed in part within TGMPA and in part flowed southward, with travel distances ranging between few kilometers and about 180 kilometers (Fig. 5, 6, 7).

Simulations carried out at three different depths (1 m, 5 m and 10 m) were in agreement in terms of overall southwards direction for all release dates considered, but differed in terms of dispersal distance. Particles released at the shallowest depth (1 m, Fig. 5) travelled at distances between few kilometers (<2–5 km) and about 100 km of coastline, while those released and travelling at 5 m (Fig. 6) and 10 m (Fig. 7) depth travelled from few kilometers (<2–5 km) to about 180 km of coastline.

### Genetic Patterns

Values of genetic diversity in all settler samples are detailed in Table 5. Similar values were observed for all diversity indices including observed (*H*_o_ = 0.617–0.692) and expected (*H*_e_ = 0.677–0.721) heterozygosity and allelic richness (AR = 7.78–9.77). All comparisons across samples using one-way ANOVA were statistically not significant (p>0.5). Only four out of 34 tests departed from HWE, but none were significant after Bonferroni correction. The software MICROCHECKER showed no evidence for scoring errors due to stuttering or large allele dropout. No linkage disequilibrium was observed between any pair of loci after Bonferroni correction. Simulations using our empirical microsatellite data in POWSIM, taking into account the sample size of each population and a wide range of predefined F_ST_ values, showed that our markers have enough statistical power to detect F_ST_ values ranging from 0.001 to 0.1 (p = 1.000).

**Table 5:** Summary of diversity indices for all *D. sargus sargus* sampling sites.

Locus	Torre Pozzella, North (N = 24)	Punta Penna Grossa, TGMPA (N = 48)	Punta Penne, South (N = 24)
**Ad5**	H_o_ = 0.790	H_o_ = 0.898	H_o_ = 0.864
	H_e_ = 0.920	H_e_ = 0.928	H_e_ = 0.921
	HWE = 0.051	HWE = 0.293	HWE = 0.057
	F_IS_ = 0.168	F_IS_ = 0.046	F_IS_ = 0.085
	AR = 14.3	AR = 14.2	AR = 15.3
**Ad86**	H_o_ = 0.737	H_o_ = 0.857	H_o_ = 0.750
	H_e_ = 0.821	H_e_ = 0.865	H_e_ = 0.861
	HWE = 0.171	HWE = 0.437	HWE = 0.475
	F_IS_ = 0.130	F_IS_ = 0.021	F_IS_ = 0.154
	AR = 7.6	AR = 9.9	AR = 9.2
**Bld9**	H_o_ = 0.818	H_o_ = 0.744	H_o_ = 0.810
	H_e_ = 0.725	H_e_ = 0.828	H_e_ = 0.818
	HWE = 0.130	HWE = 0.362	HWE = 0.945
	F_IS_ = −0.105	F_IS_ = 0.113	F_IS_ = 0.034
	AR = 5.5	AR = 7.9	AR = 7.9
**Bld15**	H_o_ = 0.833	H_o_ = 0.917	H_o_ = 0.857
	H_e_ = 0.898	H_e_ = 0.932	H_e_ = 0.905
	HWE = 0.034*	HWE = 0.118	HWE = 0.051
	F_IS_ = 0.101	F_IS_ = 0.031	F_IS_ = 0.077
	AR = 12.8	AR = 15.1	AR = 12.9
**Bld39**	H_o_ = 0.533	H_o_ = 0.667	H_o_ = 0.750
	H_e_ = 0.769	H_e_ = 0.787	H_e_ = 0.783
	HWE = 0.059	HWE = 0.114	HWE = 0.587
	F_IS_ = 0.337	F_IS_ = 0.165	F_IS_ = 0.067
	AR = 5.9	AR = 7.2	AR = 6.0
**Cld11**	H_o_ = 0.238	H_o_ = 0.238	H_o_ = 0.455
	H_e_ = 0.327	H_e_ = 0.364	H_e_ = 0.417
	HWE = 0.080	HWE = 0.037*	HWE = 1.000
	F_IS_ = 0.293	F_IS_ = 0.356	F_IS_ = −0.066
	AR = 3.0	AR = 2.7	AR = 2.9
**Cld26**	H_o_ = 0.786	H_o_ = 0.829	H_o_ = 0.895
	H_e_ = 0.880	H_e_ = 0.930	H_e_ = 0.929
	HWE = 0.492	HWE = 0.058	HWE = 0.560
	F_IS_ = 0.144	F_IS_ = 0.120	F_IS_ = 0.064
	AR = 12.0	AR = 14.7	AR = 16.3
**Cld32**	H_o_ = 0.833	H_o_ = 0.822	H_o_ = 0.761
	H_e_ = 0.790	H_e_ = 0.834	H_e_ = 0.815
	HWE = 0.586	HWE = 0.104	HWE = 0.028*
	F_IS_ = −0.002	F_IS_ = 0.025	F_IS_ = 0.090
	AR = 8.6	AR = 9.3	AR = 7.5
**Cld49**	H_o_ = 0.833	H_o_ = 0.872	H_o_ = 0.905
	H_e_ = 0.814	H_e_ = 0.849	H_e_ = 0.863
	HWE = 0.523	HWE = 0.017*	HWE = 0.519
	F_IS_ = −0.002	F_IS_ = −0.017	F_IS_ = −0.024
	AR = 9.4	AR = 9.7	AR = 11.0
**Cld93**	H_o_ = 0.222	H_o_ = 0.368	H_o_ = 0.211
	H_e_ = 0.279	H_e_ = 0.373	H_e_ = 0.189
	HWE = 0.390	HWE = 1.000	HWE = 1.000
	F_IS_ = 0.227	F_IS_ = 0.025	F_IS_ = −0.091
	AR = 2.0	AR = 2.7	AR = 2.0
**Dld14**	H_o_ = 0.783	H_o_ = 0.887	H_o_ = 0.952
	H_e_ = 0.893	H_e_ = 0.922	H_e_ = 0.916
	HWE = 0.389	HWE = 0.090	HW = 0.302
	F_IS_ = 0.146	F_IS_ = 0.050	F_IS_ = −0.015
	AR = 11.2	AR = 13.3	AR = 14.5
**Dld31**	H_o_ = 0.000	H_o_ = 0.042	H_o_ = 0.048
	H_e_ = 0.000	H_e_ = 0.041	H_e_ = 0.047
	HWE = –	HWE = 1.000	HWE = 1.000
	F_IS_ = –	F_IS_ = −0.005	F_IS_ = 0.000
	AR = 1.0	AR = 1.6	AR = 1.7
**ALL**	H_o_ = 0.617	H_o_ = 0.678	H_o_ = 0.688
	H_e_ = 0.677	H_e_ = 0.721	H_e_ = 0.705
	AR = 7.78	AR = 9.03	AR = 9.77

Details include number of individuals (N), observed (*H*_o_) and expected (*H*_e_) heterozygosity, Hardy-Weinberg equilbrium (HWE) values, F_IS_ values and allelic richness (AR). * = Non-significant after Bonferroni correction.

Comparison of allele frequencies among samples showed no significant differences in settlers at any locus or across all loci (p = 0.221). AMOVA analysis suggested no genetic sub-structuring (F_ST_ = 0.001), with no significant differences within (F_IS_ = 0.071) or among sites (F_IT_ = 0.072). All pairwise comparisons were not significant (p>0.05). The software STRUCTURE inferred one single population as most likely (K = 1; ln likelihood = −3864.8) in comparison with K = 2 (ln likelihood = −3990.1) or K = 3 (ln likelihood = −4098.6).

## Discussion

This study provides a strong indication that TGMPA is effective in protecting adults (i.e. spawners) of the white sea bream *D. sargus sargus*, accordingly with previous results [40] and the patterns observed for other Mediterranean MPAs [19], [48], [74]. In fact, TGMPA hosts the highest density and biomass of larger sized spawners across the 200 km of the sampling area along the southern Apulian Adriatic coast. Therefore, TGMPA can be considered as one of the potentially most effective sources of propagules (eggs and larvae) of white sea bream in the SW Adriatic Sea.

This hypothesis matches the findings related to a congeneric species (*Diplodus vulgaris*) that was demonstrated to have a potential egg production approx. 15 times higher within TGMPA than in outer fished areas [75].

These findings allow to hypothesize an effectively enhanced production of propagules and subsequent retention/export from TGMPA, with potential effects on population recovery/replenishment within the TGMPA and in the surrounding areas. High densities of large-sized spawners within MPAs are well known to enhance breeding stock biomass [76], favor the occurrence of spawning aggregations [77] and, consequently, generate far greater production of propagules compared to fished areas [10], [11], [78].

Even though it is reasonable to say that local populations within effective MPAs could self-sustain via propagule retention and/or seed unprotected areas via propagule export [1], empirical evidence is scarce and sometimes controversial [24], [38], [79]. Retention and/or export from a source area are mechanisms mostly driven by dispersal at the larval stage. Marine currents and long pelagic larval phases imply a high potential for long-distance dispersal in marine organisms [80]–[81]. Literature examples indicate a wide range of larval dispersal distances for fish, which may vary from a few meters to hundreds of kilometers (with an average of ∼100 km; [82]–[83]). In the case of *D. sargus sargus*, a larval dispersal of ∼100–200 km has been recently reported using otolith chemistry [84], a spatial scale which agrees with the results of the Lagrangian simulations presented here.

Although larvae may have the potential to disperse over large distances, fractions of those produced locally may be retained near the spawning areas [85]–[89]. This is generally attributed to local oceanographic structures favoring retention along with larval behavior and swimming ability, which allow larvae to swim towards or stay at the (suitable) habitats that already host the adults of the same species, sometimes contrasting sea currents [90]. Fish larvae can reach (or be retained in) suitable sites for settlement thanks to powerful sensory cues (i.e. olfactory, hearing and celestial) that are used for orientation in the pelagic environment ([91], [92] and see [93] for a review). In the present study, even though simulated particles were considered as passive, the Lagrangian simulations supported the hypothesis of propagule retention within TGMPA thanks to the presence of coastal eddies preventing a fraction of larvae to disperse far beyond the TGMPA boundaries.

It is well know, however, that larval behavior and particularly vertical migration [93] can affect dispersal pattern [94]. Therefore, the incorporation of behavioral information in Lagrangian models can deeply influence the predicted output of the models [94]–[96]. Reliable information on the pattern of vertical migration in larvae is nonetheless scarce and most Lagrangian simulation exercises considered particles as passive (see [96]). Looking at the scanty literature available on the subject, a higher rate of retention is observed whenever larval behavior is accounted for (i.e. [95], [96]). It is thus possible that our model might have underestimated larval retention at TGMPA. Further modeling and field studies will be necessary in the future to thoroughly investigate pattern, magnitude and geographical range of larval dispersal in this geographical area.

The evidence arising from the three approaches adopted in the present study (visual census, Lagrangian modeling and genetics) suggests a high degree of connectivity between the MPA and adjacent areas at the spatial scale investigated, indicative of transport of propagules well beyond the MPA boundaries. All simulations showed a general southward export, while dispersal distances varied depending on the depth of simulation. Similarly, using dispersal models in large estuarine systems of North America, other authors found a significant variability in spatial patterns of dispersed particles related to depth [97].

Lagrangian simulations and settler density observed in the field (considered as a proxy of larval production) agreed in suggesting both propagule retention and export mechanisms directed southwards. Density of settlers, moreover, did not vary significantly with distance from TGMPA, suggesting a fairly homogenous propagule dispersal in open waters over a scale of ∼100 km. On the other hand, density of settlers varied among sites, which could reflect a different suitability for settlement of coastal sites as a function of habitat type.

A significant export of larvae beyond TGMPA boundaries, as predicted by model simulations, is in agreement with the observed homogeneity found in our preliminary genetic study. Comparison of samples obtained in TGMPA and neighboring areas showed no differences in genetic variability or genetic composition, which indicates that the TGMPA is not isolated and that there is genetic connectivity among sites at the scale of approximately 50 km. The pattern of dispersion suggested by the oceanographic model could explain the genetic similarity between the TGMPA and southern sites, with down-current sites effectively replenished by TGMPA. However, the genetic homogeneity between TGMPA and northern sites cannot be explained by larval dispersal from TGMPA under the simulated oceanographic regime, as all particles originating from TGMPA either stayed within the protected area or moved southward. As a consequence, the observed genetic homogeneity could be due to gene flow from northern sites towards TGMPA and even more southern sites. In addition, a gene flow between TGMPA and adjacent northern areas could be ascribed also to the exchange of juvenile and adult individuals between TGMPA and northern sites. Evidence of connectivity among TGMPA and the adjacent northern areas has been documented only for juveniles [84]. Alternatively, as evidenced by [98]–[100], under particularly strong Sirocco (i.e. the wind blowing from the southeast) conditions the dominant southward WACC may reverse in direction resulting in a northward dispersal of larvae potentially able to homogenize the allele frequencies between TGMPA and the northern sites. From this perspective at the spatial scale investigated in the present study TGMPA does not seem to act as a genetic diversity reservoir. However this statement should be taken cautiously because the sampling scheme we adopted is not fully suitable to investigate this noteworthy point that would deserve further investigation. Additional genetic work including a large number of sampling sites and an higher number of samples in each site should be conducted to confirm the results from this preliminary study.

Gotelli [101] coined the term ‘propagule rain’ to describe the possible spatial decoupling between propagule production at spawning sites and juvenile replenishment occurring at settlement sites, due to dispersal at larval stage. Conceptually, propagule rain does not involve *a priori* any directionality in dispersal (i.e. *sensu* [17]). A number of field studies showed that abundance of fish larvae was indeed higher near MPAs and decreased, with no specific directionality, with distance from MPA boundaries [38], [102], [103]. Similar patterns were found also for mollusk settlers (used as a proxy of larval production [31], [37]). A mechanism similar to Gotelli’s propagule rain but characterized by a strong directionality might instead occur in the coastal region investigated in the present study: the results of the oceanographic simulations support the hypothesis that the specific ‘directional’ pattern observed in settlement in the SW Adriatic can be ascribed to the specific hydrodynamic conditions (e.g. current regimes) and topography of this region.

This picture does not match completely with the ‘directional’ patterns we have found in the SW Adriatic, which could be due to differences in early life-history traits of species belonging to different phyla (mollusks vs fishes), or differences in hydrodynamic conditions (e.g. current regimes) and topography (e.g. continuous coastline vs islands) among the different study areas.

Pelc et al. [17] discussed the conditions under which dispersal can be more easily detected: 1) short distance of larval dispersal from the source (e.g. a MPA), 2) directional dispersal and 3) appropriate sampling schemes. Particularly in case of a 10 km MPA with a 15-fold higher production compared to outside (as recorded for a congeneric fish at TGMPA, [75]), spatial pattern at settlement for long-dispersing species (>50 km) did not show any decline across the MPA’s boundary and over tens of kilometers. On the other hand, a large decline was modeled for short-dispersing species (i.e. <10 km) [17]. From this perspective the absence of difference among MPA and downcurrent area (i.e. South) in density of settlers could be explained by an effective dispersal of white seabream larvae, estimated to be >100 km in the study area [84]. Conversely, the difference between TGMPA and up-current area (i.e. North) in terms of settlement magnitude could be due to a reduced larval dispersal northwards as suggested by Lagrangian simulations. However, in the absence of further evidence (e.g. arising from empirical measures of larval dispersal, [22]–[24]) such an hypothesis has to be taken cautiously.

Fishes are generally characterized by relatively high larval dispersal [82] compared to mollusks. The white sea bream displays a fairly wide larval dispersal of at least 200 km along the SW Apulian coast [84]. The directional dispersal observed in the present study, the spatially replicated design used here, along with the use of multiple approaches, allowed us to obtain converging evidence about the dispersal patterns of *D. sargus sargus* around TGMPA in SW Apulia. These findings support the idea that TGMPA could produce benefits that go well beyond its boundaries.

The lack of knowledge on whether and how MPAs can benefit fisheries through propagule export remains a major roadblock to successfully designing and implementing MPAs worldwide [17], [18]. Properly assessing patterns of propagule dispersal from MPAs is crucial and the results of the present study offer useful information to build up guidelines to design both single MPAs and networks of MPAs that may satisfy both conservation and management needs. Our study also highlights the importance of integrating different approaches (field sampling, modeling and genetics; see also [104]) to investigate propagule dispersal from MPAs and get reliable convergent evidence on patterns of fish propagule dispersal.

In conclusion, the present study provides some elements suggesting that, in the case of *D. sargus sargus* in the SW Adriatic, a network of effective and self-sustaining MPAs (as it seems to be the case for TGMPA) placed at a distance of 100–200 kilometers could provide benefits for both conservation and fishery output thanks to the replenishment of fished areas through larval export. Carrying on similar studies on a number of representative species and in other areas, and possibly also taking into account propagule inflow in MPAs (e.g. using Lagrangian back-tracking techniques), will make it possible to draw more general conclusions that may help design effective regional networks of MPAs.
